# Self-calibrated pulse oximetry algorithm based on photon pathlength change and the application in human freedivers

**DOI:** 10.1117/1.JBO.28.11.115002

**Published:** 2023-11-23

**Authors:** Jingyi Wu, J. Chris McKnight, Eva-Maria S. Bønnelycke, Gerardo Bosco, Tommaso Antonio Giacon, Jana M. Kainerstorfer

**Affiliations:** a Carnegie Mellon University, Department of Biomedical Engineering, Pittsburgh, Pennsylvania, United States; b University of St Andrews, Sea Mammal Research Unit, St Andrews, United Kingdom; c University of Padua, Department of Biomedical Sciences, Padua, Italy; d Padua University Hospital, Institute of Anesthesia and Intensive Care, Padua, Italy; e University of Padua, Department of Medicine, Padua, Italy; f Carnegie Mellon University, Neuroscience Institute, Pittsburgh, Pennsylvania, United States

**Keywords:** self-calibrated algorithm, pulse oximetry, oxygen saturation, photon pathlength, near-infrared spectroscopy, human freediving

## Abstract

**Significance:**

Pulse oximetry estimates the arterial oxygen saturation of hemoglobin (${\mathrm{SaO}}_{2}$) based on relative changes in light intensity at the cardiac frequency. Commercial pulse oximeters require empirical calibration on healthy volunteers, resulting in limited accuracy at low oxygen levels. An accurate, self-calibrated method for estimating ${\mathrm{SaO}}_{2}$ is needed to improve patient monitoring and diagnosis.

**Aim:**

Given the challenges of calibration at low ${\mathrm{SaO}}_{2}$ levels, we pursued the creation of a self-calibrated algorithm that can effectively estimate ${\mathrm{SaO}}_{2}$ across its full range. Our primary objective was to design and validate our calibration-free method using data collected from human subjects.

**Approach:**

We developed an algorithm based on diffuse optical spectroscopy measurements of cardiac pulses and the modified Beer–Lambert law (mBLL). Recognizing that the photon mean pathlength (⟨L⟩) varies with ${\mathrm{SaO}}_{2}$ related absorption changes, our algorithm aligns/fits the normalized ⟨L⟩ (across wavelengths) obtained from optical measurements with its analytical representation. We tested the algorithm with human freedivers performing breath-hold dives. A continuous-wave near-infrared spectroscopy probe was attached to their foreheads, and an arterial cannula was inserted in the radial artery to collect arterial blood samples at different stages of the dive. These samples provided ground-truth ${\mathrm{SaO}}_{2}$ via a blood gas analyzer, enabling us to evaluate the accuracy of ${\mathrm{SaO}}_{2}$ estimation derived from the NIRS measurement using our self-calibrated algorithm.

**Results:**

The self-calibrated algorithm significantly outperformed the conventional method (mBLL with a constant ⟨L⟩ ratio) for ${\mathrm{SaO}}_{2}$ estimation through the diving period. Analyzing 23 ground-truth ${\mathrm{SaO}}_{2}$ data points ranging from 41% to 100%, the average absolute difference between the estimated ${\mathrm{SaO}}_{2}$ and the ground truth ${\mathrm{SaO}}_{2}$ is 4.23%±5.16% for our algorithm, significantly lower than the 11.25%±13.74% observed with the conventional approach.

**Conclusions:**

By factoring in the variations in the spectral shape of ⟨L⟩ relative to ${\mathrm{SaO}}_{2}$, our self-calibrated algorithm enables accurate ${\mathrm{SaO}}_{2}$ estimation, even in subjects with low ${\mathrm{SaO}}_{2}$ levels.

## 1 Introduction

Oxygen level in the arterial blood (${\mathrm{SaO}}_{2}$), namely the percentage of oxygen-bound hemoglobin out of the total hemoglobin, is one of the most important health indicators in patient care. Its precise measurement requires an arterial blood draw to be processed by a blood gas analyzer but in clinics or home settings, it is often estimated noninvasively with a finger pulse oximeter, which is denoted by ${\mathrm{SpO}}_{2}$ (“p” indicates pulse oximetry).^1^ The underlying principle behind pulse oximetry is diffuse optical spectroscopy—by shining light at two wavelengths (usually red and infrared) into the finger and detecting the intensity changes from the heart pulsation, ${\mathrm{SpO}}_{2}$ can be calculated by utilizing the fact that light at different wavelengths is absorbed differently based on the amount of oxygen-bound hemoglobin.^2^

Analytically, ${\mathrm{SpO}}_{2}$ can be calculated by the modified Beer–Lambert Law (mBLL), which relates the absorption of light by the tissue to changes in hemoglobin concentration and the pathlength traveled by the light. In this method, conventionally, the mean pathlength (⟨L⟩) or the ⟨L⟩ ratio between two wavelengths is also assumed to be constant.^3^ Although ⟨L⟩ should not be constant, because analytically it is a function of the absorption ($\mu_{a}$) and reduced scattering ($\mu_{s}^{\prime }$) coefficient of tissue,^3^^,^^4^ for finger pulse oximeters, this is accounted for by empirical calibration and assumption that the pathlength does not change over small changes in saturation level. During the calibration process, healthy adult volunteers inhale air with lower oxygen content to decrease their ${\mathrm{SaO}}_{2}$. Calibration factors derived from the measured signals are then used to correlate with each ${\mathrm{SaO}}_{2}$ level.^2^^,^^4^

One drawback of the empirical calibration process is that ${\mathrm{SaO}}_{2}$ of the healthy volunteers can only be lowered to around 80%, because a lower value could be harmful to the subjects. As a result, obtaining calibration factors for ${\mathrm{SaO}}_{2}<80\%$ in humans is particularly challenging. Thus the accuracy of the finger pulse oximeters is high at high ${\mathrm{SaO}}_{2}$, but it decreases significantly as ${\mathrm{SaO}}_{2}$ decreases.^2^^,^^4^^,^^5^

Low ${\mathrm{SaO}}_{2}$ levels can be found in the various situations, such as acute respiratory distress syndrome,^6^ chronic obstructive pulmonary disease,^7^ asthma,^8^ COVID-19 infections,^9^^,^^10^ fetuses during delivery,^11^^,^^12^ marine mammals,^13^ or human freedivers during prolonged apnea.^14^ In these subjects, accurate measurement for ${\mathrm{SaO}}_{2}<80\%$ is important and could guide vital therapeutic decisions. To provide a potential solution to this problem and aim to improve the patient or study outcomes, we developed a self-calibrated algorithm that can estimate ${\mathrm{SaO}}_{2}$ at all saturation levels, which in its core, does not assume constant $\mu_{a}$ or constant ⟨L⟩ across ${\mathrm{SaO}}_{2}$ levels.

We examined the application of this algorithm on breath-holding human freedivers, whose ${\mathrm{SaO}}_{2}$ dropped to as low as 41% during dives to a depth of 42 m in a protocol similar to what was reported by Bosco et al.^15^ By comparing the ${\mathrm{SpO}}_{2}$ calculated by our algorithm and the conventional method (mBLL with constant ⟨L⟩ ratio) to the ground truth ${\mathrm{SaO}}_{2}$ from blood gas measurements, we found that the accuracies are comparable at high ${\mathrm{SaO}}_{2}$ levels, but our algorithm significantly outperformed the conventional method at low ${\mathrm{SaO}}_{2}$ levels. These findings demonstrated the potential of our self-calibrated algorithm in accurately estimating ${\mathrm{SaO}}_{2}$ levels across a wide range of conditions, particularly in situations where conventional method yields inaccurate result or empirical calibration is not applicable.

## 2 Method

### 2.1 Theoretical Frameworks: Self-Calibrated Method for ${\mathrm{SpO}}_{2}$ Calculation

In this section, we outline the formulation of our self-calibrated algorithm designed for ${\mathrm{SpO}}_{2}$ calculation. The central premise is that ⟨L⟩ is a function of $\mu_{a}$ and $\mu_{s}^{\prime }$, and given that $\mu_{a}$ depends on the saturation level, ⟨L⟩ also varies accordingly.

In the following discussion, we use the term “⟨L⟩ ratio” to denote the ratio ${\left\langle L\right\rangle }^{\lambda_{n}}/{\left\langle L\right\rangle }^{\lambda_{1}}$, where $\lambda_{n}$ represents NIRS measurements at the n’th (n=1,2,3,…) wavelength. Thus when multiple wavelengths (at least two) are used, the ⟨L⟩ ratio describes a spectral shape across these wavelengths with the value at the first wavelength being 1. In contrast to common notation in pulse oximetry, where red and infrared wavelengths are typically used and ⟨L⟩ ratio is usually represented by ${\left\langle L\right\rangle }^{\text{infrared}}/{\left\langle L\right\rangle }^{\text{red}}$, which is a singular value.

Building on this foundation, if we obtain a measured ⟨L⟩ ratio from experiment that is ${\mathrm{SpO}}_{2}$ dependent, and we also have its analytical representation that depends on ${\mathrm{SpO}}_{2}$, it becomes feasible to align or fit the analytical ⟨L⟩ ratio with the measured version to deduce ${\mathrm{SpO}}_{2}$. Our self-calibrated algorithm thus consists of three main components: (1) constructing a measured ⟨L⟩ ratio from the continuous-wave near-infrared spectroscopy (CW-NIRS) measurement using mBLL, (2) constructing an analytical ⟨L⟩ ratio derived from the analytical equation of ⟨L⟩, and (3) determining the ${\mathrm{SpO}}_{2}$ value that best matches the measured and analytical ⟨L⟩ ratios.

#### 2.1.1 Constructing the measured ⟨L⟩ ratio

The arterial oxygen saturation, ${\mathrm{SaO}}_{2}$, is defined as ${\mathrm{SaO}}_{2}=[\mathrm{HbO}]/([\mathrm{HbO}]+[\mathrm{Hb}]$), where [HbO] and [Hb] are the concentrations of oxy- and deoxy-hemoglobin, respectively, in the arterial blood. If we assume that the measured light intensity is solely modulated by the artery blood volume variations during the cardiac cycle, leading to changes in [HbO] and [Hb] at the heartrate (HR) frequency, then the change in optical density (ΔOD) can be defined by the mBLL:^3^

$$\Delta {\mathrm{OD}}^{\lambda }=\ln(\frac{I_{d}^{\lambda }}{I_{s}^{\lambda }})={\left\langle L\right\rangle }^{\lambda }\Delta \mu_{a}^{\lambda }={\left\langle L\right\rangle }^{\lambda }(\Delta [\mathrm{HbO}]\varepsilon_{\mathrm{HbO}}^{\lambda }+\Delta [\mathrm{Hb}]\varepsilon_{\mathrm{Hb}}^{\lambda }),$$ (1)

where $I_{d}$ and $I_{s}$ are the intensities measured at the diastolic and systolic states of the cardiac cycle, respectively; $\Delta \mu_{a}$ is the change in the absorption coefficient; and ε is the extinction coefficient.

${\mathrm{SpO}}_{2}$ can be calculated by ${\mathrm{SpO}}_{2}=\Delta [\mathrm{HbO}]/(\Delta [\mathrm{HbO}]+\Delta [\mathrm{Hb}])$, substituting this into Eq. (1) and with measurements at multiple wavelengths ($\lambda_{n}$, n=1,2,3,…), Eq. (1) can be rearranged as

$${\left\langle L\right\rangle }^{\lambda_{n}}=\Delta {\mathrm{OD}}^{\lambda_{n}}{\left\{\Delta [\mathrm{HbO}][\varepsilon_{\mathrm{HbO}}^{\lambda_{n}}+({\mathrm{SpO}}_{2}^{-1}-1)\varepsilon_{\mathrm{Hb}}^{\lambda_{n}}]\right\}}^{-1}.$$ (2)

Then the measured ⟨L⟩ ratio, denoted by ⟨L^⟩measuredλ, is defined as

⟨L^⟩measuredλ(SpO2)=⟨L⟩λn⟨L⟩λ1=ΔODλnΔODλ1εHbOλ1+(SpO2−1−1)εHbλ1εHbOλn+(SpO2−1−1)εHbλn. (3)

From Eq. (3), we can see that the ratio ${\Delta \mathrm{OD}}^{\lambda_{n}}/{\Delta \mathrm{OD}}^{\lambda_{1}}$ from measurement inherently possesses a spectral shape across wavelengths. However, varying ${\mathrm{SpO}}_{2}$ values in the equation can alter this shape, leading to changes in ⟨L^⟩measuredλ in relation to ${\mathrm{SpO}}_{2}$.

#### 2.1.2 Constructing the analytical ⟨L⟩ ratio

The analytical equation of ⟨L⟩ in semi-infinite medium for a reflectance measurement is^16^

$${\left\langle L\right\rangle }_{\text{analytical}}^{\lambda }\equiv {\mathrm{DPF}}_{\text{seminf}}^{\lambda }\cdot r=\frac{\sqrt{3\mu_{s}^{\prime \lambda }}}{2\sqrt{\mu_{a}^{\lambda }}}\frac{r\sqrt{3\mu_{a}^{\lambda }\mu_{s}^{\prime \lambda }}}{r\sqrt{3\mu_{a}^{\lambda }\mu_{s}^{\prime \lambda }}+1}\cdot r=\frac{3}{2}\frac{r^{2}\mu_{s}^{\prime \lambda }}{r\sqrt{3\mu_{a}^{\lambda }\mu_{s}^{\prime \lambda }}+1},$$ (4)

where ${\mathrm{DPF}}_{\text{seminf}}$ is the differential pathlength factor in semi-infinite medium, and r is the source–detector distance.

In Eq. (4), $\mu_{a}^{\lambda }$ can be substituted by $\mu_{a}^{\lambda }=[\mathrm{HbO}]\varepsilon_{\mathrm{HbO}}^{\lambda }+[\mathrm{Hb}]\varepsilon_{\mathrm{Hb}}^{\lambda }=[\mathrm{HbO}][\varepsilon_{\mathrm{HbO}}^{\lambda }+({\mathrm{SpO}}_{2}^{-1}-1)\varepsilon_{\mathrm{Hb}}^{\lambda }]$, making ${\left\langle L\right\rangle }_{\text{analytical}}^{\lambda }$ a function of ${\mathrm{SpO}}_{2}$. With that, the analytical ⟨L⟩ ratio, ⟨L^⟩analyticalλ, is defined as

⟨L^⟩analyticalλ(SpO2)=⟨L⟩analyticalλn(μaλ(SpO2))⟨L⟩analyticalλ1(μaλ(SpO2)). (5)

Similar to ⟨L^⟩measuredλ from Eq. (3), the spectral shape of ⟨L^⟩analyticalλ will also change in relation to ${\mathrm{SpO}}_{2}$.

#### 2.1.3 ${\mathrm{SpO}}_{2}$ determination by aligning the measured and analytical ⟨L⟩ ratios

With ⟨L^⟩measuredλ and ⟨L^⟩analyticalλ defined in Eqs. (3) and (5), respectively, our objective is to find the ${\mathrm{SpO}}_{2}$ value that will best align the spectral shapes of these two ratios. To achieve this, we quantify the similarity between ⟨L^⟩measuredλ and ⟨L^⟩analyticalλ using the residual sum of squares (RSS) error (RSS=Σλ[⟨L^⟩measuredλ−⟨L^⟩analyticalλ]2) for the full range of ${\mathrm{SpO}}_{2}$ (0% to 100%). The ${\mathrm{SpO}}_{2}$ value yielding the minimum RSS provides our best estimate for true arterial oxygen saturation (${\mathrm{SaO}}_{2}$). Here we chose an iterative approach rather than a direct fitting approach for demonstration purpose, but computational efficiency can be improved if needed.

**Fig. 1:**
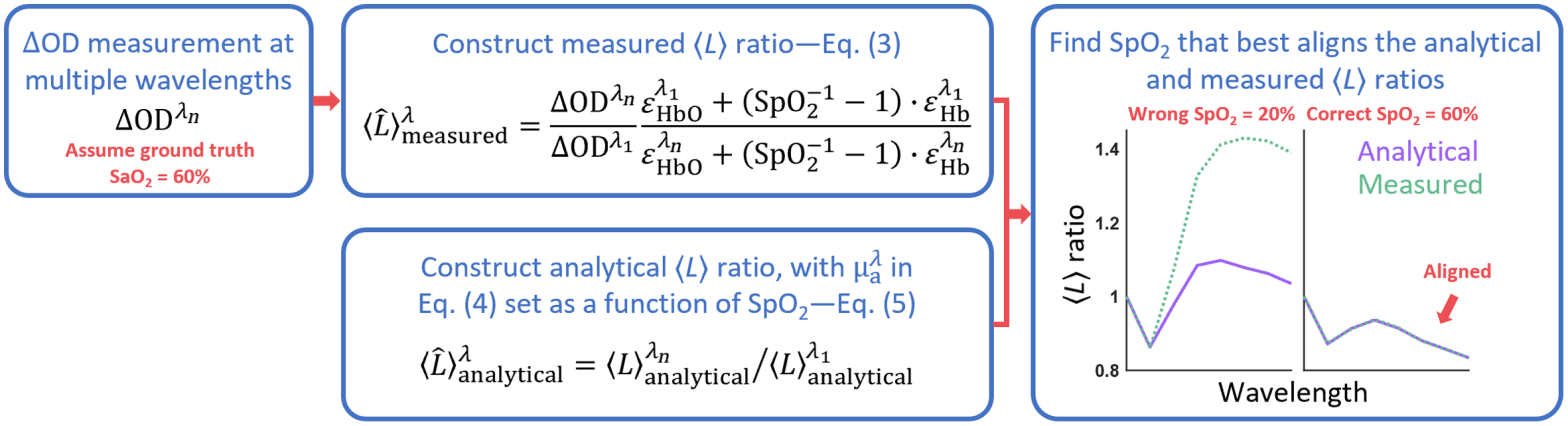
Flowchart of the self-calibrated pulse oximetry algorithm.

To illustrate the implementation of the self-calibrated algorithm, we provide a flowchart in Fig. 1. In this example, we assume the ground truth ${\mathrm{SaO}}_{2}$ to be 60%. As indicated in the rightmost box, an accurate estimation of ${\mathrm{SaO}}_{2}$ (with ${\mathrm{SpO}}_{2}=60\%$) results in spectral shapes of the measured and analytical ⟨L⟩ ratios that closely resemble each other. It is important to note that this is only a visual representation—the actual values of ⟨L⟩ ratios and their shapes across wavelengths will vary based on the specific experiment.

### 2.2 Algorithm Validation with Monte-Carlo Simulation

To validate the self-calibrated algorithm, we used Monte-Carlo photon simulation (MCX Lab)^17^ to generate synthetic data. To simulate cardiac pulsations, simulations at systolic and diastolic $\mu_{a}$ were performed. We modeled a semi-infinite homogenous media with ${\mathrm{SaO}}_{2}$ at 20%, 40%, 60%, 80%, and 100% at eight wavelengths (740, 760, 780, 800, 820, 840, 860 and 880 nm). A 2% change from baseline total hemoglobin concentration ([HbT], assumed to be 50  μM) due to pulsation was assumed. Reduced scattering $\mu_{s}^{\prime }$ was assumed to be constant across ${\mathrm{SpO}}_{2}$ levels and pulsation. In ${\mathrm{cm}}^{-1}$, $\mu_{s}^{\prime }$ was calculated using $\mu_{s}^{\prime }=a\cdot \lambda^{b}$, where λ is in nm, and a=260.7 and b=−0.4668 were obtained from the literature for human subdermal tissue.^12^^,^^18^ For example, at 800 nm and ${\mathrm{SaO}}_{2}=100\%$, $\mu_{s}^{\prime }=11.51\;{\mathrm{cm}}^{-1}$ and $\mu_{a}=0.0930$ and $0.0949\;{\mathrm{cm}}^{-1}$ at diastolic and systolic states, respectively. The dimensions of the modeled media were set to be 350, 350, and 200 mm for its width, length, and height, respectively. A simulated isotropic source with a photon count of $5\times 10^{8}$ was placed at the center of the modeled tissue surface, and intensities emitted from the surface (as a reflectance measurement) at a source–detector distance of 3 cm were recorded. The simulated intensities at the systolic ($I_{d}$) and diastolic ($I_{s}$) states were then converted to ΔOD by $\ln(I_{d}/I_{s})$.

Using the simulated ΔOD, we first calculated ${\mathrm{SpO}}_{2}$ using the conventional method:^11^

$${\mathrm{SpO}}_{2}=\frac{\varepsilon_{\mathrm{HbO}}^{\lambda_{2}}R({\left\langle L\right\rangle }^{\lambda_{2}}/{\left\langle L\right\rangle }^{\lambda_{1}})-\varepsilon_{\mathrm{Hb}}^{\lambda_{1}}}{\left(\varepsilon_{\mathrm{HbO}}^{\lambda_{1}}-\varepsilon_{\mathrm{Hb}}^{\lambda_{1}})-R({\left\langle L\right\rangle }^{\lambda_{2}}/{\left\langle L\right\rangle }^{\lambda_{1}})(\varepsilon_{\mathrm{HbO}}^{\lambda_{2}}-\varepsilon_{\mathrm{Hb}}^{\lambda_{2}}\right)},$$ (6)

where $R={\Delta \mathrm{OD}}^{\lambda_{1}}/{\Delta \mathrm{OD}}^{\lambda_{2}}$, and the ⟨L⟩ ratio ${\left\langle L\right\rangle }^{\lambda_{2}}/{\left\langle L\right\rangle }^{\lambda_{1}}$ is assumed to be constant across ${\mathrm{SpO}}_{2}$ levels and subjects. Here the constant ⟨L⟩ ratio was taken from the literature, which suggests that the differential pathlength factor (DPF=⟨L⟩/r) is a function of age and wavelength^19^: $\mathrm{DPF}=223.3+0.05624\cdot \text{age}-5.723\cdot 10^{-7}\cdot \lambda^{3}+0.001245\cdot \lambda^{2}-0.9025\cdot \lambda$. Given an age of 30, $\lambda_{1}=760\;\mathrm{nm}$, $\lambda_{2}=840\;\mathrm{nm}$, r=3  cm, and ⟨L⟩=DPF·r, we calculated ${\left\langle L\right\rangle }^{840\;\mathrm{nm}}/{\left\langle L\right\rangle }^{760\;\mathrm{nm}}=0.87$. In addition, to demonstrate how different assumptions of this constant ⟨L⟩ ratio values could influence of ${\mathrm{SpO}}_{2}$ calculations, we considered a second ratio of 0.61. We then applied the self-calibrated algorithm to the same data, comparing its results with those of the conventional method.

### 2.3 ${\mathrm{SpO}}_{2}$ Extraction Using Two Versus Eight Wavelengths in the Presence of Noise in ΔOD

As described in Sec. 2.2, we simulated ΔOD over eight wavelengths, all of which were incorporated into the self-calibrated algorithm. In the human freediving experiment (detailed in the next section), however, data were only available for two wavelengths—760 and 840 nm. Recognizing that real experiments introduce noise into the ΔOD, we aimed to assess the impact of noise on ${\mathrm{SpO}}_{2}$ extraction when using two versus eight wavelengths with the self-calibrated algorithm.

To examine this, we began with the noise-free simulated ΔOD at r=3  cm and introduced Gaussian white noise [with standard deviation (STD) of 0.002] to ΔOD values at each wavelength (740, 760, 780, 800, 840, 860, and 880 nm). We then input the noisy ΔOD either at all eight wavelengths or just the two (760 and 840 nm) to the self-calibrated algorithm to derive the ${\mathrm{SpO}}_{2}$. This entire procedure was repeated 100 times, each with a unique Gaussian white noise introduced at every wavelength. Subsequently, we calculated the mean and STD of the extracted ${\mathrm{SpO}}_{2}$ values for comparison.

### 2.4 Data Collection from Human Freediving Experiment

To evaluate the algorithm on human data, we analyzed measurements from breath-hold divers. The study’s experimental protocol received approval from the University of Padua’s Department of Biomedical Science Human Ethical Committee and followed the guidelines established by the declaration of Helsinki. Prior to their participation in the study, all subjects provided their written informed consent.

The freediving experiment was conducted in the 42 m deep indoor thermal pool “Y-40 THE DEEP JOY” in, Padua, Montegrotto, Italy. For each experimental trial, the participant diver completed a 15 m deep breath-hold dive and then recovered at the surface before completing a 42 m deep breath-hold dive. The descent and ascent of all dives were completed with the assistance of a weighted sled, meaning the divers experienced minimal physical exertion. The start, bottom, and end time of each dive was recorded. In this study, nine subjects performed sled-assisted dives. NIRS measurement and blood draws were carried out on these subjects during seven dives of 15 m and nine dives of 42 m.

**Fig. 2:**
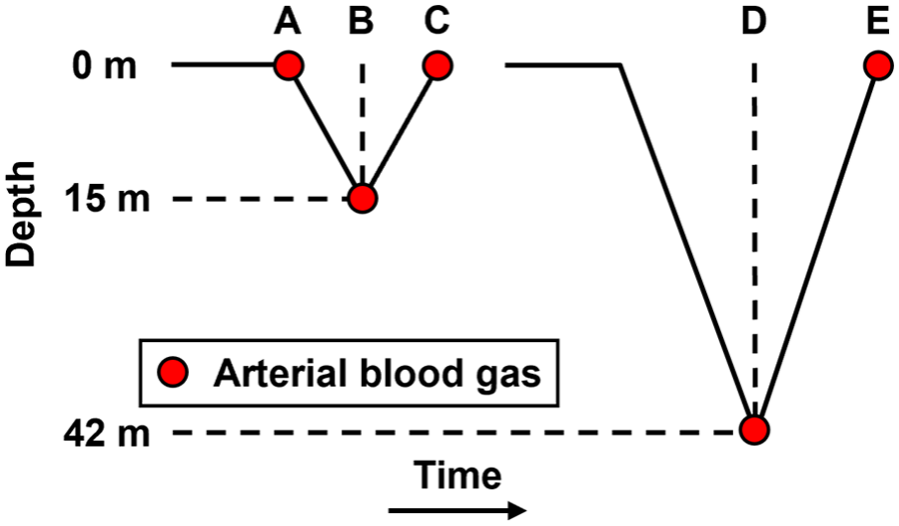
Illustration for 15 and 42 m dives and arterial blood extraction time points.

An arterial cannula was inserted in the radial artery of each diver’s nondominant arm as described by Bosco et al.^15^ and Paganini et al.^20^ for underwater blood sample collection at different stages of the dive as shown in Fig. 2: A (15 m start), B (15 m bottom), C (15 m end), D (42 m bottom), and E (42 m end). There was no blood sample collection at the start of the 42 m dives to limit the amount of blood taken from the divers. All the blood samples used for this study were taken while the divers’ heads were submerged under water (i.e., the predescent blood sample for the 15 m dive (point A) was taken right after the divers’ submerged under water and began their breath-hold, and the end-ascent blood samples (points C and E) were taken when the divers’ surfaced and before they took their first breath). The approximate timings for all the blood draws were recorded. A blood gas analyzer that uses CO-Oximetry (Stat Profile Prime Plus, Nova Biomedical Italia S.r.l., Lainate, Italy) was used for obtaining the ${\mathrm{SaO}}_{2}$ on site.

In addition, CW-NIRS measurements were taken during the dives using a waterproof device, PortaDiver, which was developed from the PortaLite mini (Artinis, Medical System BV, Netherlands).^14^ The sensor contains three light-emitting diodes (3, 3.5, and 4 cm source–detector distance) with two wavelengths each (760 and 840 nm) and one photodiode with ambient light protection. It operates at a sampling rate of 10 Hz and was placed on the diver’s forehead and secured by a swimming cap.

### 2.5 Application of Self-Calibrated Algorithm and Conventional Method on Freediving Data

First, we calculated ${\mathrm{SpO}}_{2}$ using the conventional method with a constant ⟨L⟩ ratio of 0.87 derived from the literature (see Sec. 2.2).^19^ Specifically, we applied the mBLL [Eq. (1)] to the measured OD data at 760 and 840 nm from the CW-NIRS device to obtain time traces of Δ[HbO] and Δ[Hb]. In MATLAB (Mathworks, Inc., Natick, Massachusetts, United States), a third-order zero-phase Butterworth high-pass filter at 1 Hz was applied to the Δ[HbO] and Δ[Hb] time traces. Spectrograms of Δ[HbO] and Δ[Hb] were then generated with a segment length of 10 s and an overlapping percentage of 99%. From these spectrograms, we identified the HR time trace using MATLAB’s “tfridge” function. Finally, ${\mathrm{SpO}}_{2}$ time traces were calculated by ${\mathrm{SpO}}_{2}=\Delta [\mathrm{HbO}](\mathrm{HR})/(\Delta [\mathrm{HbO}](\mathrm{HR})+\Delta [\mathrm{Hb}](\mathrm{HR}))$, and a 5-s moving average was applied to smooth the time trace.

For the self-calibrated algorithm, we first extracted the ΔOD time trace at the HR with the spectrogram and “tfridge” function. We then input ΔOD at each time point for two wavelengths (760 and 840 nm) into Eq. (3) to calculate the measured ⟨L⟩ ratio. The same $\mu_{a}$ (as a function of ${\mathrm{SpO}}_{2}$) and $\mu_{s}^{\prime }$ used in Sec. 2.2 were employed to calculate the analytical ⟨L⟩ ratio. Finally, ${\mathrm{SpO}}_{2}$ at each time point was derived by minimizing the RSS (Σλ[⟨L^⟩measuredλ−⟨L^⟩analyticalλ]2) between the measured and analytical ⟨L⟩ ratio as described in Sec. 2.1.

After obtaining the ${\mathrm{SpO}}_{2}$ time traces from both the self-calibrated algorithm and the conventional method, we compared them to the ${\mathrm{SaO}}_{2}$ measurements, which were regarded as the ground truth, from the blood gas analyzer. To account for the uncertainties in the recorded blood draw timings, we averaged the ${\mathrm{SpO}}_{2}$ around ±2  s of the recorded timepoints and then used these values to compare to the ${\mathrm{SaO}}_{2}$. In our analysis, we first plotted ${\mathrm{SpO}}_{2}$ against ${\mathrm{SaO}}_{2}$ for both methods. This was complemented with Bland–Altman analysis to demonstrate the accuracy of ${\mathrm{SpO}}_{2}$ extraction for each method. Furthermore, we divided our data based on ${\mathrm{SaO}}_{2}$ levels—high (>90%) and low (<90%). For each category, we assessed the ${\mathrm{SpO}}_{2}$ extraction performance by calculating the absolute difference between ${\mathrm{SpO}}_{2}$ and ${\mathrm{SaO}}_{2}$.

As shown in Fig. 2, ${\mathrm{SaO}}_{2}$ was extracted at time points A, B, and C at 15 m dives and at E and F for 42 m dives. All 9 subjects completed the 42 m dive with ideally 9×2=18 data points expected from two blood gas measurements per dive. However, two subjects had unreliable data due to very noisy signals, likely from probe movement under the swimming cap. This resulted in a reduction to 14 data points. Additionally, two data points had missing information in the measurement log, further reducing the count to 12 data points for the 42 m dives. 7 out of the 9 subjects completed the 15 m dive, with ideally 7×3=21 data points expected from three blood gas measurements per dive. Noisy signals in three measurements reduced this to 18 data points. One missing entry in the measurement log further reduced the final count to 11 data points for the 15 m dives. In summary, we have 12 data points from the 42 m dives and 11 from the 15 m dives, totaling 23 data points for the ${\mathrm{SaO}}_{2}$ and ${\mathrm{SpO}}_{2}$ comparison. When categorized by ${\mathrm{SaO}}_{2}$ levels, we had 10 data points for ${\mathrm{SaO}}_{2}<90\%$ and 13 for ${\mathrm{SaO}}_{2}>90\%$.

## 3 Results

### 3.1 Validation of the Self-Calibrated Algorithm with Simulated Data

**Fig. 3:**
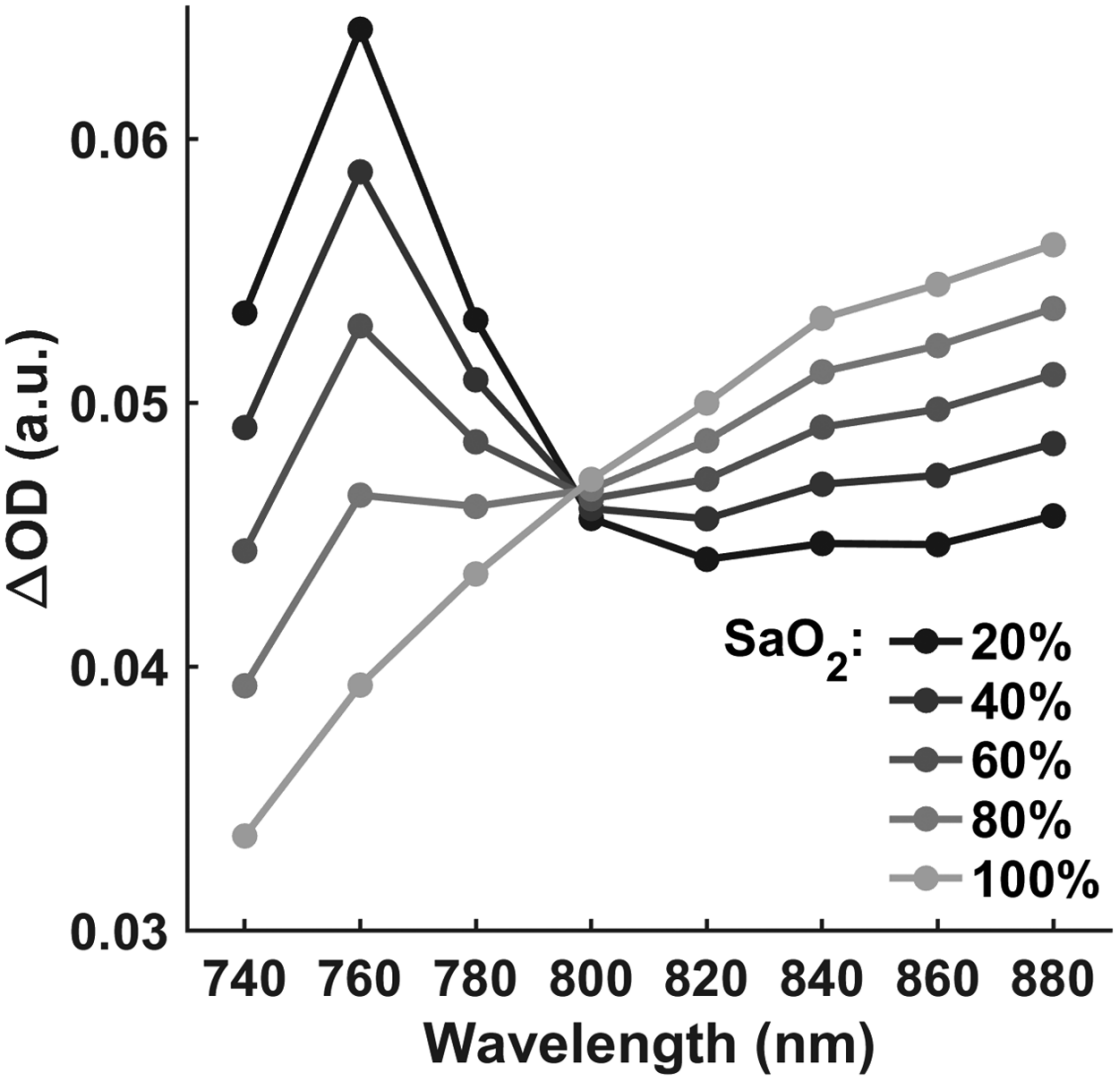
Simulated ΔOD versus wavelength at ${\mathrm{SaO}}_{2}=20\%$ to 100%.

In Monte-Carlo simulation with MCX Lab, we simulated changes in $\mu_{a}$ corresponding to the systolic and diastolic phases of cardiac pulsation. The simulated ΔOD ranging from ${\mathrm{SaO}}_{2}=20\%$ to 100% is shown in Fig. 3. As expected, the spectral shape of ΔOD changes with ${\mathrm{SaO}}_{2}$ levels.

**Fig. 4:**
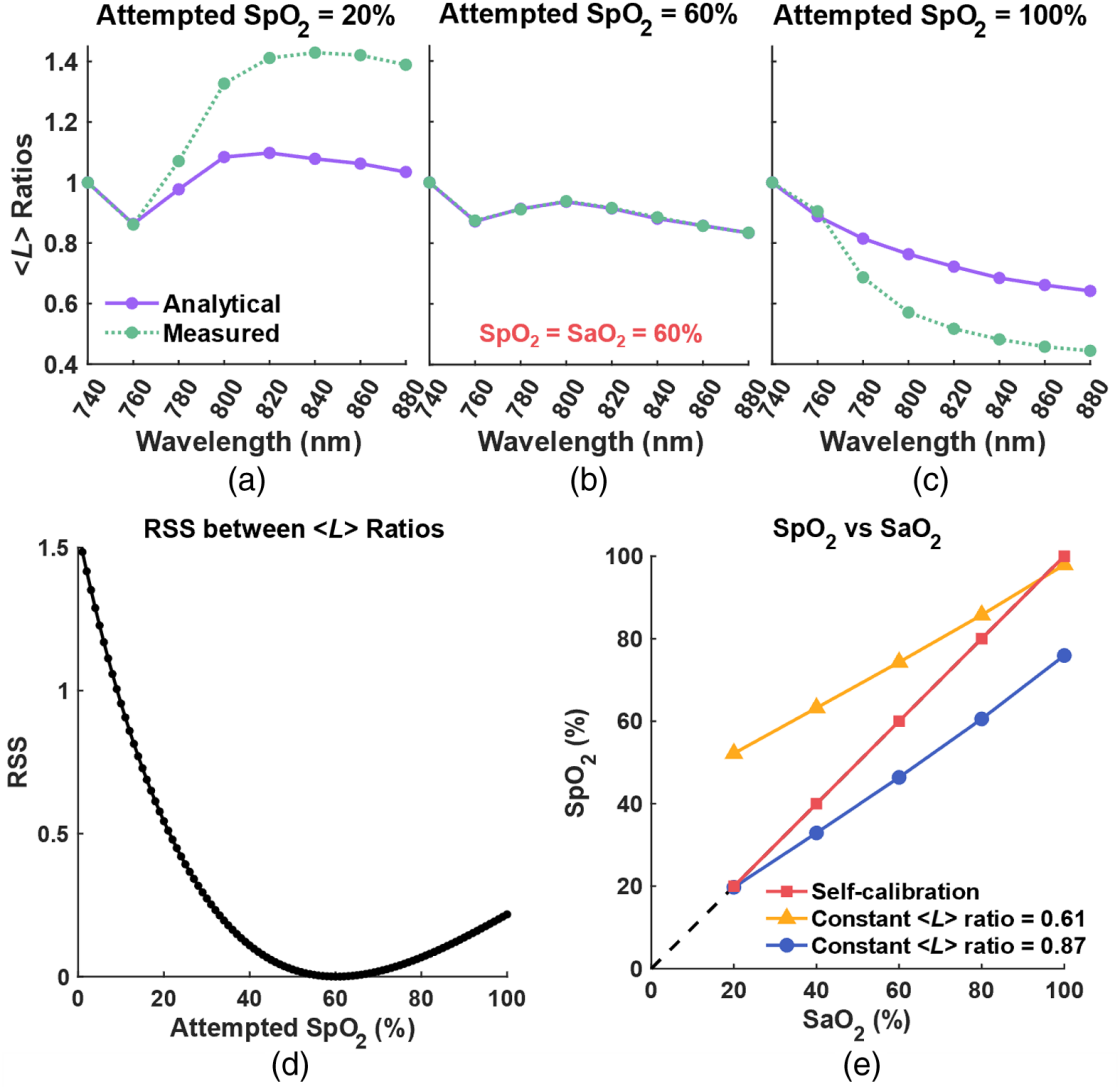
(a)–(c) Comparison between analytical (solid purple) and measured (dotted green) ⟨L⟩ ratio at ${\mathrm{SpO}}_{2}=20\%$, 60%, and 100%, respectively. (d) RSS between analytical and measured ⟨L⟩ ratio at ${\mathrm{SpO}}_{2}=0\%$ to 100%. (e) Comparison between ${\mathrm{SpO}}_{2}$ calculated by the self-calibrated algorithm (red square) and the conventional method with constant ⟨L⟩ ratio = 0.61 (yellow triangle) and 0.87 (blue circle).

Taking the simulated ΔOD at ${\mathrm{SaO}}_{2}=60\%$ as an example, we applied both our algorithm (optimizing for ⟨L⟩ ratio changes) and the conventional method (with constant ⟨L⟩ ratio) as described previously. For our algorithm, Figs. 4(a)–4(c) represent input ${\mathrm{SpO}}_{2}$ at 20%, 60%, and 100%, respectively. The spectral shapes of analytical and measured ⟨L⟩ ratio closely overlap when ${\mathrm{SpO}}_{2}$ reaches ${\mathrm{SaO}}_{2}=60\%$. By iterating ${\mathrm{SpO}}_{2}$ values from 0% to 100% in the algorithm, we plotted the RSS (Σλ[⟨L^⟩measured(λ)−⟨L^⟩analytical(λ)]2) between the analytical and measured ⟨L⟩ ratio. The RSS versus ${\mathrm{SpO}}_{2}$ in Fig. 4(d) shows a U-shape with its minimum error at the expected ${\mathrm{SpO}}_{2}=60\%$. In Fig. 4(e), we show all the extracted ${\mathrm{SpO}}_{2}$ values from our algorithm (red squares) and those from the conventional method—Eq. (6) with constant ⟨L⟩ ratios (${\left\langle L\right\rangle }^{840\;\mathrm{nm}}/{\left\langle L\right\rangle }^{760\;\mathrm{nm}}$) of 0.61 (yellow triangles) and 0.87 (blue circles).

In Fig. 4(e), we observe that the ${\mathrm{SpO}}_{2}$ values derived from our algorithm accurately follow the unity line. ${\mathrm{SpO}}_{2}$ values calculated by the conventional method, however, depends on the manually set constant ⟨L⟩ ratios. When a ratio of 0.61 (yellow triangle) was used, the calculated ${\mathrm{SpO}}_{2}$ values are relatively close to the unity line at higher ${\mathrm{SaO}}_{2}$ levels (80% to 100%). However, the accuracy diminishes for lower saturation levels. In contrast, when using a ratio of 0.87 (blue circles) taken from the literature, the extracted ${\mathrm{SpO}}_{2}$ values align more closely to the unity line at lower ${\mathrm{SaO}}_{2}$ levels, particularly around 20%.

It is curial to clarify that the ratio of 0.87, taken from the literature, does not generally ensure enhanced ${\mathrm{SpO}}_{2}$ extraction accuracy at lower saturation levels. This specific value was derived from measurements on the frontal human head, which consists of various tissue layers that combine artery, venous, and capillary components.^19^ In contrast, our simulations utilized a semi-infinite rectangular slab volume, a geometry distinctly different from the human head’s structure. As we will demonstrate in the next section, the ratio of 0.87 is quite adequate for measurement taken from the human forehead, resulting in good ${\mathrm{SpO}}_{2}$ extraction accuracy at higher saturation levels. These findings thus highlight the importance of carefully tailoring the ⟨L⟩ ratio within the mBLL to the specific context and application.

### 3.2 ${\mathrm{SpO}}_{2}$ Extraction Using Two Versus Eight Wavelengths in the Presence of Noise in ΔOD

**Fig. 5:**
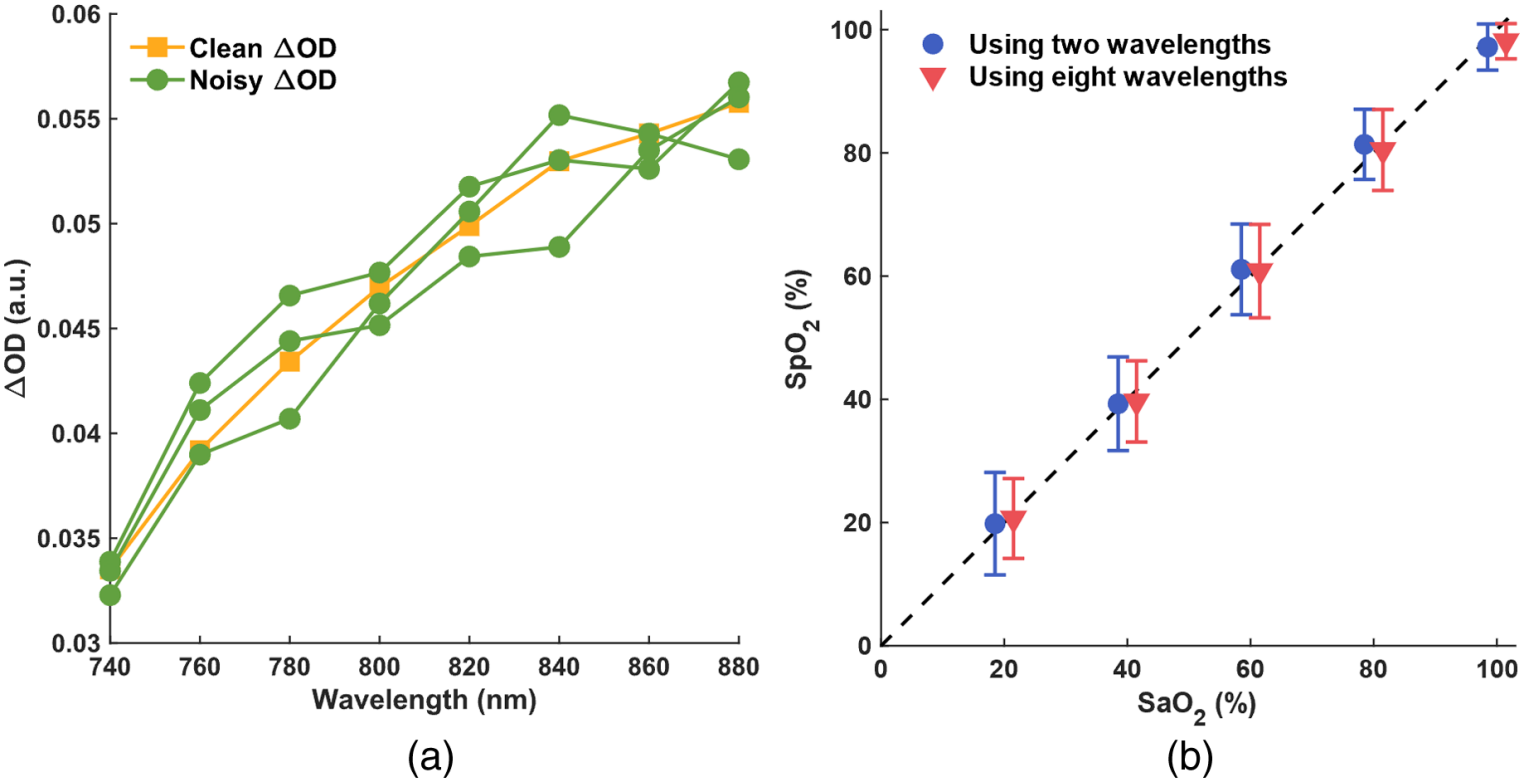
(a) Clean ΔOD at ${\mathrm{SaO}}_{2}=100\%$ (yellow square) and three examples of noisy ΔOD (green circle). (b) ${\mathrm{SpO}}_{2}$ extraction results from the self-calibrated algorithm using two (blue circle) versus eight (red triangle) wavelengths.

In Sec. 2.3, we described the process of adding Gaussian white noise to simulated noise-free ΔOD across eight wavelengths. We then processed these noisy ΔOD values using either two (760 and 840 nm) or all eight wavelengths with the self-calibrated algorithm to compute ${\mathrm{SpO}}_{2}$. Figure 5(a) shows an example comparison between the clean ΔOD at ${\mathrm{SaO}}_{2}=100\%$ (yellow squares) and three noisy ΔOD examples (green circles).

For simulated ΔOD at each ${\mathrm{SaO}}_{2}$ value (20%, 40%, 60%, 80%, and 100%), we processed 100 distinct noisy ΔOD sets through the self-calibrated algorithm, leading to 100 ${\mathrm{SpO}}_{2}$ estimations per ${\mathrm{SaO}}_{2}$ level. Figure 5(b) shows the mean and STD of these ${\mathrm{SpO}}_{2}$ values. To enhance visual clarity, we slightly offset the datasets representing results from two and eight wavelengths on the x axis. We can see that the ${\mathrm{SpO}}_{2}$ results, both in terms of mean and STD, from two (blue circles) and eight (red triangles) wavelengths are notably similar and align closely with the unity line. This similarity indicates that with the self-calibrated algorithm, accurate ${\mathrm{SaO}}_{2}$ estimation is feasible using just two wavelengths, as will be demonstrated in the subsequent human freediving experiment.

### 3.3 Application of the Self-Calibrated Algorithm and Conventional Method to Freediver Data

**Fig. 6:**
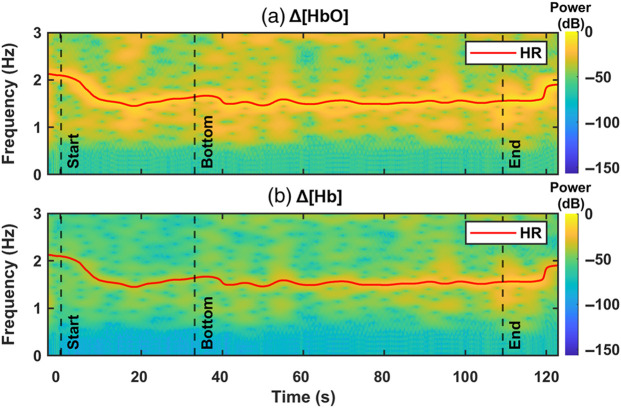
(a), (b) Spectrogram for Δ[HbO] and Δ[Hb] with HR time trace (red line).

**Fig. 7:**
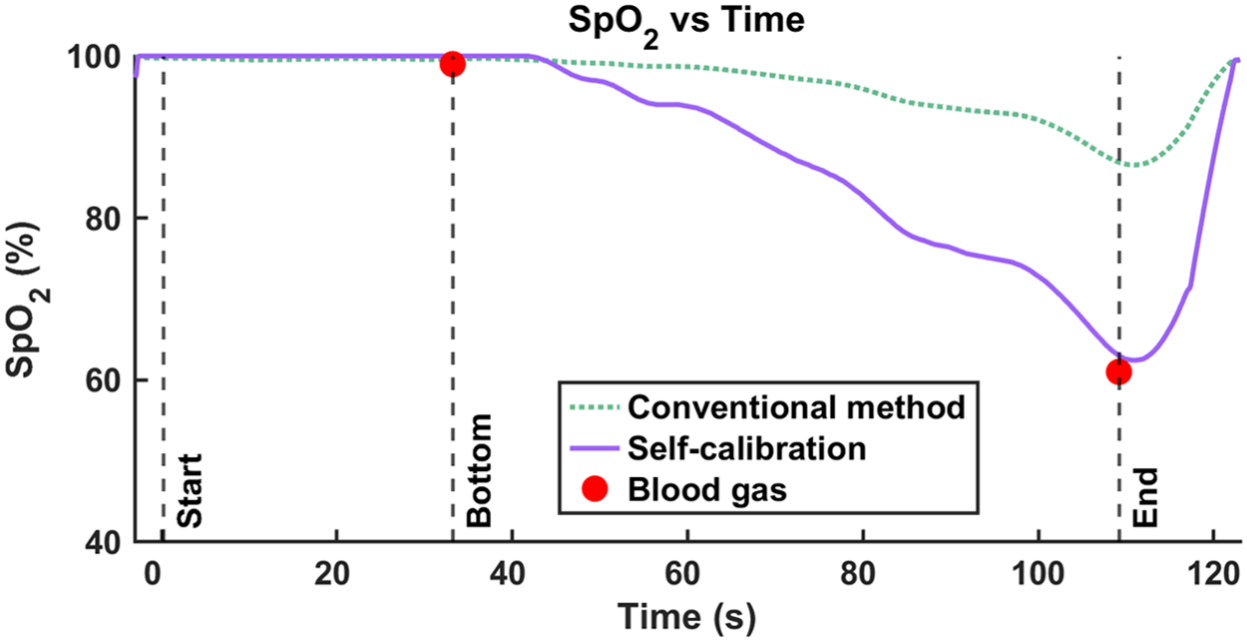
Comparison between ${\mathrm{SpO}}_{2}$ extracted from self-calibrated algorithm (solid purple line) and the conventional method (dotted green line) to ground truth ${\mathrm{SaO}}_{2}$ from blood gas (red circles).

Following the conventional approach to calculate ${\mathrm{SpO}}_{2}$, we took the measured data at r=3  cm and calculated the Δ[HbO] and Δ[Hb] time traces using mBLL as described in Sec. 2.4, with the assumption of a constant ⟨L⟩ ratio (${\left\langle L\right\rangle }^{840\;\mathrm{nm}}/{\left\langle L\right\rangle }^{760\;\mathrm{nm}}=0.87$). An example result from a 42 m dive is presented in Figs. 6(a) and 6(b), which show example spectrograms for Δ[HbO] and Δ[Hb] with their identified HR (red solid line). ${\mathrm{SpO}}_{2}$ time trace—${\mathrm{SpO}}_{2}=\Delta [\mathrm{HbO}](\mathrm{HR})/(\Delta [\mathrm{HbO}](\mathrm{HR})+\Delta [\mathrm{Hb}](\mathrm{HR}))$—is depicted in Fig. 7. Comparing the ${\mathrm{SpO}}_{2}$ time trace (green dotted line) to the blood gas measurement (red circles), we observed that the calculated ${\mathrm{SpO}}_{2}$ of 99.6% at 42 m (“bottom”) is consistent with the measured ${\mathrm{SaO}}_{2}$ of 99%. However, at the end of the 42 m dive, near the surface, the calculated ${\mathrm{SpO}}_{2}$ of 87.8% largely overestimates the ground truth ${\mathrm{SaO}}_{2}$ of 61% (a 27.8% difference).

For the self-calibrated algorithm, we extracted ΔOD at the HR from the spectrograms for each wavelength (760 and 840 nm) and input them to the algorithm using the same data. The purple solid line in Fig. 7 shows the ${\mathrm{SpO}}_{2}$ time traces calculated by the algorithm. The ${\mathrm{SpO}}_{2}$ calculations are consistent with the ground-truth ${\mathrm{SaO}}_{2}$ measured from the blood gas (red circles) not only at 42 m bottom, where ${\mathrm{SpO}}_{2}$ is 100% and ${\mathrm{SaO}}_{2}$ is 99%, but also at the surface, where ${\mathrm{SpO}}_{2}$ is 63.2% and ${\mathrm{SaO}}_{2}$ is 61% (a 2.2% difference).

**Fig. 8:**
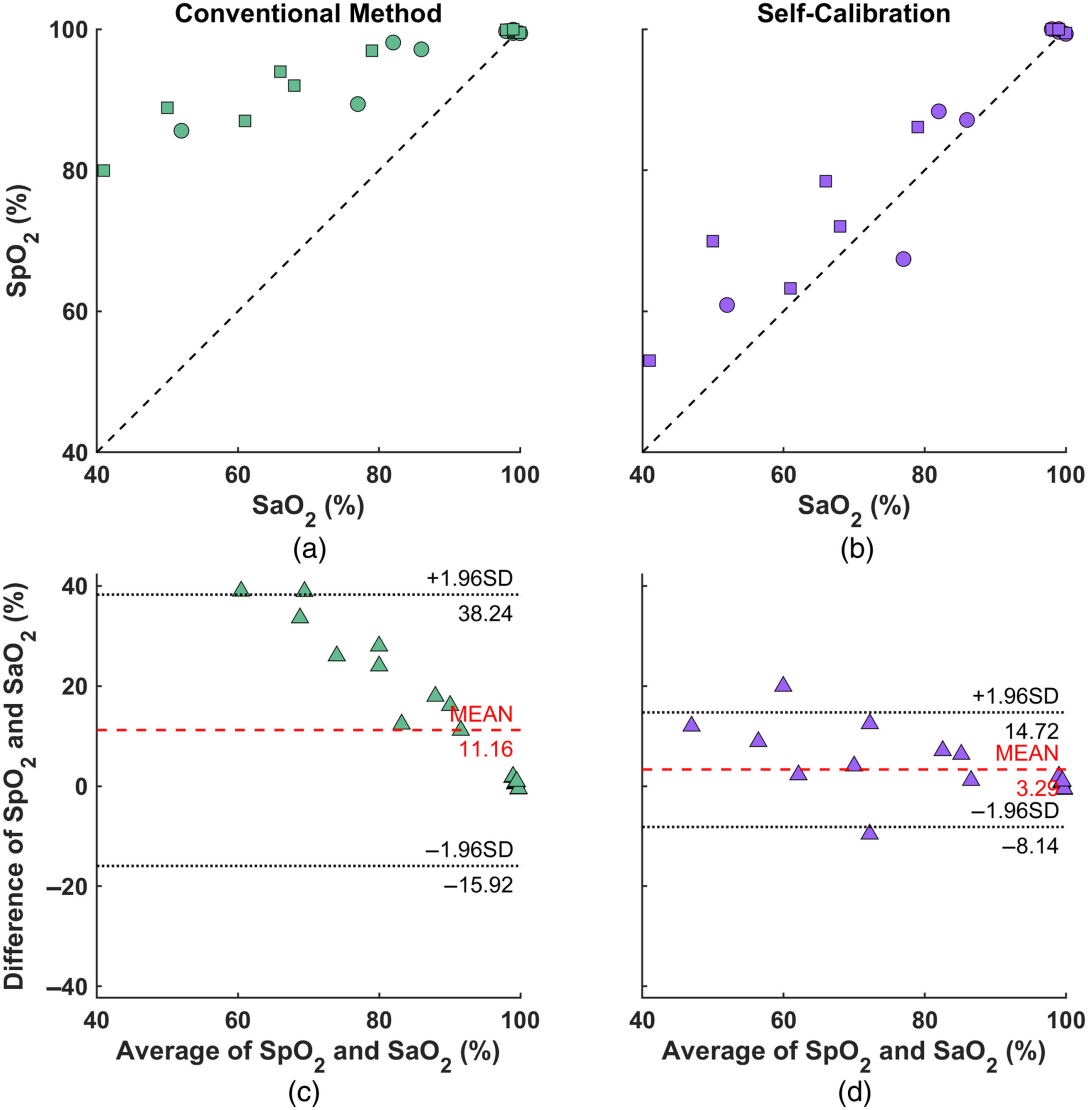
${\mathrm{SpO}}_{2}$ versus ${\mathrm{SaO}}_{2}$ for all data points: (a) conventional method and (b) self-calibrated algorithm. Data from 15 and 42 m dives are marked by circles and squares, respectively. (c), (d) Bland–Altman plots for (a) and (b), respectively.

In Figs. 8(a) and 8(b), we plotted ${\mathrm{SpO}}_{2}$ against ${\mathrm{SaO}}_{2}$ for all the data. For the self-calibrated algorithm, the ${\mathrm{SpO}}_{2}$ values predominantly fall in line with the unity line (represented by the black dashed line). In contrast, the conventional method often overestimates ${\mathrm{SaO}}_{2}$, particularly at lower saturation levels.

This difference becomes more evident in the Bland–Altman plots of Figs. 8(c) and 8(d). The self-calibrated algorithm exhibits a modest average bias of 3.29% between ${\mathrm{SpO}}_{2}$ and ${\mathrm{SaO}}_{2}$, with most of the data points clustering near this central bias line. Additionally, the limits of agreement, capturing 95% of the differences between ${\mathrm{SpO}}_{2}$ and ${\mathrm{SaO}}_{2}$, are narrow (−8.14% to 14.72%). Importantly, the residuals show no systematic biases, confirming the reliability of this method.

In contrast, the conventional method presents a substantially higher average bias of 11.16% between ${\mathrm{SpO}}_{2}$ and ${\mathrm{SaO}}_{2}$. The limits of agreement for this method are also broader (−15.92% to 38.24%), signifying greater variability. Moreover, a noticeable trend in the residuals reveals an increased bias at lower saturation levels.

Taken together, these findings demonstrate the precision and consistency of our self-calibrated algorithm, indicating its superior performance in estimating ${\mathrm{SpO}}_{2}$ across various saturation levels compared to the conventional method.

**Fig. 9:**
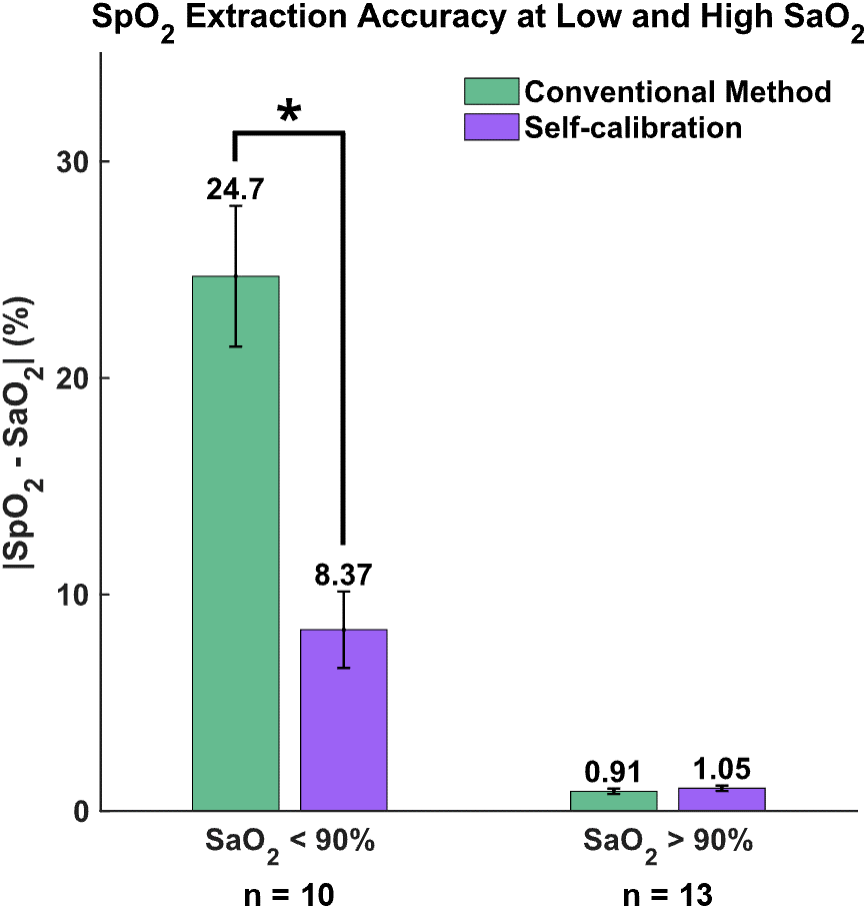
Absolute difference between the extracted ${\mathrm{SpO}}_{2}$ and ground-truth ${\mathrm{SaO}}_{2}$ at low (<90%) and high (>90%) saturation levels. *p<0.05.

To further evaluate the robustness of the two methods under different saturation levels, we divided our dataset based on ${\mathrm{SaO}}_{2}$ levels into two categories: high (>90%) and low (<90%). The metric used to quantify performance was the absolute difference between ${\mathrm{SpO}}_{2}$ and ${\mathrm{SaO}}_{2}$ ($\left|{\mathrm{SpO}}_{2}-{\mathrm{SaO}}_{2}\right|$). Figure 9 illustrates the mean and standard error of these differences for each method.

For high ${\mathrm{SaO}}_{2}$ levels (>90%), both methods perform similarly well, with differences of 0.91% for the conventional method and a slightly higher 1.05% for the self-calibrated algorithm. However, the distinction between the two methods becomes pronounced at lower ${\mathrm{SaO}}_{2}$ levels (<90%): the self-calibrated algorithm shows a discrepancy of only 8.37%, whereas the conventional method has a 24.7% difference. The statistical significance of this variation was further demonstrated by the Wilcoxon rank sum test with the significance level was set to be 0.05.

These results emphasize the superior accuracy of the self-calibrated algorithm, particularly when the ${\mathrm{SaO}}_{2}$ is low, when compared to the conventional approach.

## 4 Discussion

Commercial pulse oximeters are known to suffer from decreased accuracy at lower saturation levels due to limitations in empirical calibration. On the other hand, when applying mBLL to calculate ${\mathrm{SpO}}_{2}$, the conventional method assumes a constant ⟨L⟩ ratio between wavelengths. This also causes inaccuracy in ${\mathrm{SaO}}_{2}$ estimation, because the change in ⟨L⟩ ratio might be negligible when ${\mathrm{SaO}}_{2}$ is high and within a narrow range (e.g., 90% to 100%), but it increases drastically as ${\mathrm{SaO}}_{2}$ decreases. To address this, we introduced a self-calibrated algorithm that calculates ${\mathrm{SpO}}_{2}$ by accounting for the changes in ⟨L⟩ ratio.

This new approach was first validated with MC simulations. After that, it was tested on human freediving data, comparing the results to the ground truth ${\mathrm{SaO}}_{2}$ obtained from arterial blood samples. Our findings, as illustrated in Figs. 7–9, show that while both the conventional and self-calibrated methods perform similarly at high saturation levels, the self-calibrated method performs significantly better at low saturation levels.

In the literature, various methods have been proposed to estimate DPF (represented as ⟨L⟩/r) for CW-NIRS applications. Talukdar et al.^21^ applied the extended Kalman filter for real-time ΔDPF estimation to enhance Δ[HbO] and Δ[Hb] calculations, although requiring a prior DPF value. This approach adjusts for ΔDPF without gauging its absolute value. For the absolute estimation, Huang and Hong^22^ introduced dual square-root cubature Kalman filters with a multichannel probe. However, this requires an initial phantom experiment calibration, and their method was only applied on simulated data. While both have merits, their feasibility for ${\mathrm{SpO}}_{2}$ calculations remains uncertain. In another study, Yossef Hay et al.^23^ introduced a calibration-free finger pulse oximeter using two close wavelengths (761 and 818 nm) to mitigate the variability of the ⟨L⟩ difference between these wavelengths. Although they achieved good agreement at higher saturations (>90%), its performance at lower levels remains unspecified. A shared attribute among these three methods and our self-calibrated algorithm, however, is the fundamental assumption of a semi-infinite and homogenous medium.

In addition to estimating ${\mathrm{SaO}}_{2}$ with high accuracy across various saturation levels, the self-calibrated algorithm has the potential to improve ${\mathrm{SaO}}_{2}$ estimation in patients with darker skin tones. This is a particularly relevant issue, as recent discussions have highlighted the reduced ${\mathrm{SaO}}_{2}$ estimation accuracy at lower saturations, especially in patients with darker skin, which became a significant problem during COVID-19 pandemic.^9^^,^^24^ In the self-calibrated algorithm, the spectral shape of $\mu_{a}$ plays a crucial role, as it is currently a function of ${\mathrm{SpO}}_{2}$. Based on the established extinction coefficients, melanin, oxy-hemoglobin, and deoxy-hemoglobin have distinct absorption spectra. Thus by incorporating an additional $\mu_{a}$ spectra as a function of melanin concentrations, our algorithm could potentially account for differences in skin tone, leading to improved estimation accuracy across diverse patient populations.

In addition, further improvements and studies are needed to confirm the reliability of our algorithm in other research and clinical settings. First, the analytical equation for ⟨L⟩ we used in the algorithm is for semi-infinite media with reflectance setup. Most human tissues, however, are multilayered. It will be necessary to study how layered tissue (or skull thickness in this experiment) influences the ${\mathrm{SpO}}_{2}$ calculation. An improved formulation of the self-calibrated algorithm with the two-layer mBLL by Hiraoka et al.^25^ or with the analytical partial pathlength by García et al.^26^ might be necessary. Second, we assumed that the light attenuation only comes from change in $\mu_{a}$ due to heart pulsation, and $\mu_{s}^{\prime }$ being constant over ${\mathrm{SaO}}_{2}$ and heart pulsation. Based on these assumptions, we used the same [HbT], change in [HbT] from baseline (2%), and $\mu_{s}^{\prime }$ (taken from literature) in the algorithm for all subjects. With skin melanin concentration, skull thickness, etc. taken into consideration, these assumptions are inaccurate. In order to overcome the assumption of baseline optical properties, a frequency-domain NIRS could be used to provide the exact optical properties of the specific measurement site of the subject.^16^ Further validation using data from various clinical settings and patient populations would be beneficial in assessing the generalizability of our findings. Finally, same as other CW-NIRS measurements, our method also suffers from measurement noise such as motion artifacts, especially because the NIRS device (PortaDiver) we used was held down by a swimming cap. We used a Butterworth high-pass filter in MATLAB to remove the low-frequency noise and then extracted ΔOD(t) at HR from spectrograms. Processing our signal with other conventional or deep learning-based methods^27^^,^^28^ could potentially improve the ${\mathrm{SpO}}_{2}$ calculation by the self-calibrated algorithm. Additionally, the motion artifacts in the measurements could be reduced through improved attachment methods.

In conclusion, the proposed self-calibrated algorithm demonstrates improved performance in ${\mathrm{SaO}}_{2}$ estimation compared to the conventional method of using mBLL with constant ⟨L⟩ ratio. By optimizing the spectral shape of the optical pathlength and accounting for differences in skin tone, our algorithm has the potential to improve the accuracy of ${\mathrm{SpO}}_{2}$ estimation across a diverse range of patient populations. Future research should focus on further validating the algorithm in different clinical scenarios and exploring its potential applications in other diagnostic and monitoring contexts, such as calculation of cerebral metabolic rate of oxygen, organ saturation map, transabdominal fetal pulse oximetry, and others.

## Data Availability

Data and code are available at 10.1184/R1/24530353.
